# Solvent-mediated extraction of disperse dyes from polyester: correlating Cyrene extraction yields with molecular topological and chemical descriptors

**DOI:** 10.1039/d5ra08950f

**Published:** 2026-01-15

**Authors:** Philip Fernando, Andrew Hebden, Chenyu Du, Parikshit Goswami

**Affiliations:** a Technical Textiles Research Centre, University of Huddersfield Queensgate Huddersfield HD1 3DH UK Philip.fernando@hud.ac.uk; b School of Applied Sciences, University of Huddersfield Queensgate Huddersfield HD1 3DH UK

## Abstract

The desorption of disperse dyes from synthetic textiles remains a critical challenge in sustainable textile processing, particularly when targeting structurally diverse dye classes. This study examines the structure–property relationships governing solvent-mediated dye extraction using Cyrene™, focusing on azo and anthraquinone systems. Dye removal was conducted over three successive cycles under optimised conditions. Statistical analysis (ANOVA and Tukey's HSD) revealed that dye type, cycle number, and their interaction significantly influenced removal efficiency. Three of the four dyes exhibited statistically similar mean reductions, while CI Disperse Blue 56 (DB), the most planar and least topologically complex, showed the lowest efficiency. Topological descriptors derived from ChemDraw and Chem3D modelling identified DB as the smallest in molecular dimensions, whereas CI Disperse Red 60, CI Disperse Yellow 114, and CI Disperse Orange 30 were more structurally intricate. Correlation analyses (Pearson's and Spearman's) yielded limited predictive relationships, though descriptors such as log *P* and log *S* showed relatively higher coefficients. Despite the absence of definitive correlations, dyes with greater surface area and molecular complexity demonstrated enhanced interaction with Cyrene™, achieving up to 98.5% colour reduction. These findings underscore the robustness of the developed method and suggest that molecular architecture plays a contributory role in solvent–dye interactions under controlled conditions.

## Abstract 2

Process overview: PET textiles are cut, subjected to solvent-mediated dye extraction, and dye removal efficiency is evaluated using computational analysis.

## Graphical abstract

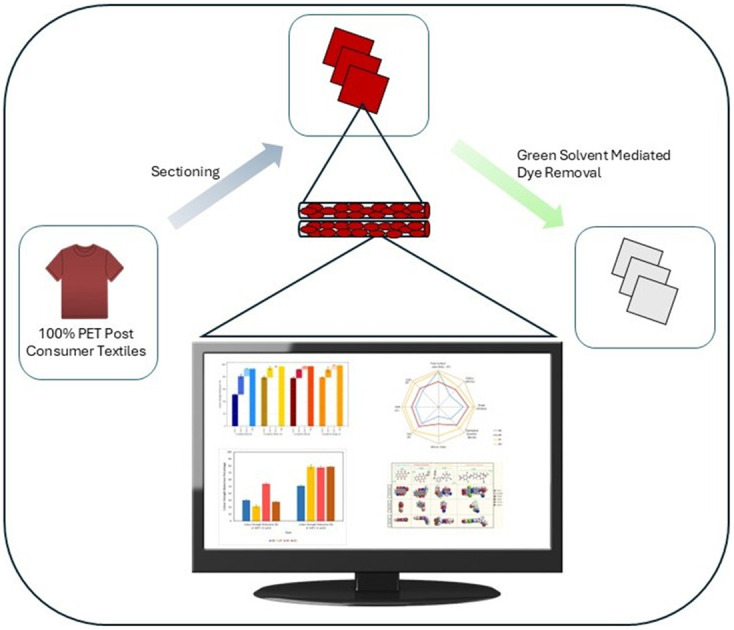

## 1 Introduction

The global textile industry has undergone significant transformation over the past decades, with synthetic fibres now dominating production landscapes. Polyethylene terephthalate (PET) fibres constitute 59% of global fibre production as of 2024, fundamentally altering the dyeing processes and environmental considerations within the sector.^1^ This shift towards synthetic materials has necessitated the widespread adoption of disperse dyes, particularly anthraquinone and azo systems, which collectively account for over 75% of disperse dye applications due to their exceptional fastness properties and strong affinity for synthetic substrates.^2,3^

The molecular characteristics that make disperse dyes effective for synthetic fibre dyeing simultaneously present considerable challenges for end-of-life textile processing. The inherently low water solubility of these dyes, combined with their reduced chemical potential when bound to PET fibres, creates a thermodynamically unfavourable environment for dye removal.^2,4^ This molecular stability, whilst advantageous during the textile's useable lifetime, becomes problematic during fibre-to-fibre recycling processes, where residual dyes can compromise the molecular weight and intrinsic viscosity of recycled polymers.^5^ Furthermore, incomplete dye removal results in colour contamination of recycled fibres, limiting their commercial viability and perpetuating the linear consumption model that the industry seeks to transcend.^6^

Current methodologies for dye removal from textile substrates broadly encompass two strategic approaches: destructive degradation and extractive separation.^7^ Destructive methods, including photodegradation and oxidative treatments, whilst effective in eliminating dyes, often result in the formation of potentially harmful by-products and represent a loss of valuable chemical resources.^8–10^ Extractive approaches, conversely, offer the potential for dye recovery and reuse, thereby supporting circular economy principles.^4,11^ Within extractive methodologies, back-stripping techniques attempt dye removal from intact fabrics, whilst alternative approaches involve fabric depolymerisation followed by dye extraction from the resulting solution.^12^

Existing extractive technologies, however, present significant limitations that impede their widespread industrial adoption. Conventional solvent systems often require harsh chemicals such as carriers and polar aprotic solvents, which pose environmental and safety concerns.^12–15^ Energy–intensive processes, exemplified by supercritical carbon dioxide extraction, demand substantial capital investment and operational costs.^16,17^ Oxidative treatments, whilst effective, may compromise fibre integrity and generate environmentally persistent compounds.^18,19^ These limitations highlight the critical need for sustainable alternatives that can achieve comparable extraction efficiencies whilst adhering to green chemistry principles.^20^

The emergence of bio-based solvents represents a promising avenue for addressing these challenges. Green solvents, defined as environmentally friendly solvents or bio-solvents derived from the processing of agricultural crops, offer sustainable alternatives to conventional chemical systems.^21^ Cyrene™ (dihydrolevoglucosenone), derived from cellulose waste streams, exemplifies a new generation of sustainable solvents that align with green chemistry principles. In textile recycling, the removal of disperse dyes from PET fibres is a critical prerequisite for producing high-quality, dye-free feedstock suitable for closed-loop processes. Employing Cyrene™ for solvent-mediated dye extraction not only addresses this technical challenge but also enhances the environmental performance of textile recycling systems by reducing reliance on hazardous chemicals and supporting circular economy objectives.^22–25^ Its biodegradable nature, low toxicity profile, and unique molecular structure suggest potential for effective dye–solvent interactions.^26,27^ However, despite its recognised potential in extraction and catalytic processes, the systematic investigation of Cyrene's efficacy in textile dye removal remains limited.

**Fig. 1:**
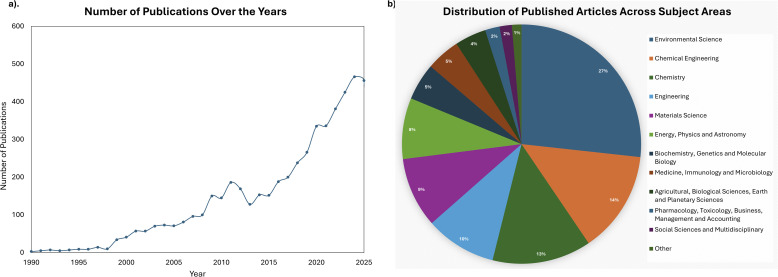
Trends in research on dye removal from textiles: (a) annual number of publications over time, and (b) distribution of published articles across different subject areas.

The growing significance of this research area is evidenced by the increasing number of publications indexed in the SCOPUS database, as illustrated in Fig. 1a, which demonstrates rising academic and industrial interest in textile dye removal from both natural and synthetic fabrics. This research encompasses dye removal from fabrics, dye effluents, and wastewater from textile industries, spanning diverse disciplines including environmental science, chemical engineering, materials science, and chemistry (Fig. 1b). Despite this growing interest, a fundamental knowledge gap exists in understanding the molecular–level interactions between Cyrene and structurally diverse disperse dyes.

The establishment of structure–property relationships between dye molecular characteristics and solvent-mediated removal efficiency represents a critical step towards predictive frameworks for sustainable textile processing.^28,29^ Such relationships would enable the rational design of extraction processes, optimisation of solvent selection, and development of targeted approaches for specific dye classes. Furthermore, a quantitative understanding of these interactions would provide the scientific foundation necessary to transition green chemistry—defined as the approach that reduces pollution at its source by minimising or eliminating the hazards of chemical feedstocks, reagents, solvents, and products—from conceptual aspiration to practical industrial application.^20^

This investigation addresses these challenges by systematically evaluating Cyrene as a solvent for disperse dye removal from textile substrates, with particular focus on anthraquinone and azo dye systems. Through controlled extraction experiments and statistical validation of molecular descriptor correlations, this study aims to establish quantitative relationships between dye structure and removal efficiency. The outcomes will contribute to the development of predictive models for solvent–dye interactions, supporting the transition towards sustainable textile processing methodologies whilst providing the industry with scientifically grounded tools for implementing circular economy principles in textile manufacturing.

## 2 Experimental

### 2.1 Materials

Four commercially available anthraquinone and azo disperse dyes were selected for this investigation: CI Disperse Red 60 (DR), CI Disperse Blue 56 (DB), CI Disperse Yellow 114 (DY), and CI Disperse Orange 30 (DO), all supplied by Regency FCB UK Ltd. The textile substrate consisted of plain weave PET fabric with an area density of 78 g m^−2^, sourced from Whaleys Fabrics, Bradford, UK. Chemical reagents, including acetic acid, sodium acetate, sodium carbonate, and sodium dithionite, were obtained from Sigma-Aldrich and used as received without further purification. The levelling agent Univadine TOP was procured from Town End (Leeds) plc, whilst the detergent Ultravon JUN was supplied by Huntsman Textile Effects, Singapore. All dyeing and extraction procedures were performed using a Roaches Pyrotec 3 laboratory dyeing machine. The bio-based solvent Cyrene™ (dihydrolevoglucose-none) was obtained from Circa Group for the dye removal experiments, with deionised water employed as the co-solvent system.

### 2.2 Methods

#### 2.2.1. HTHP dyeing of fabrics for dye removal

**Fig. 2:**
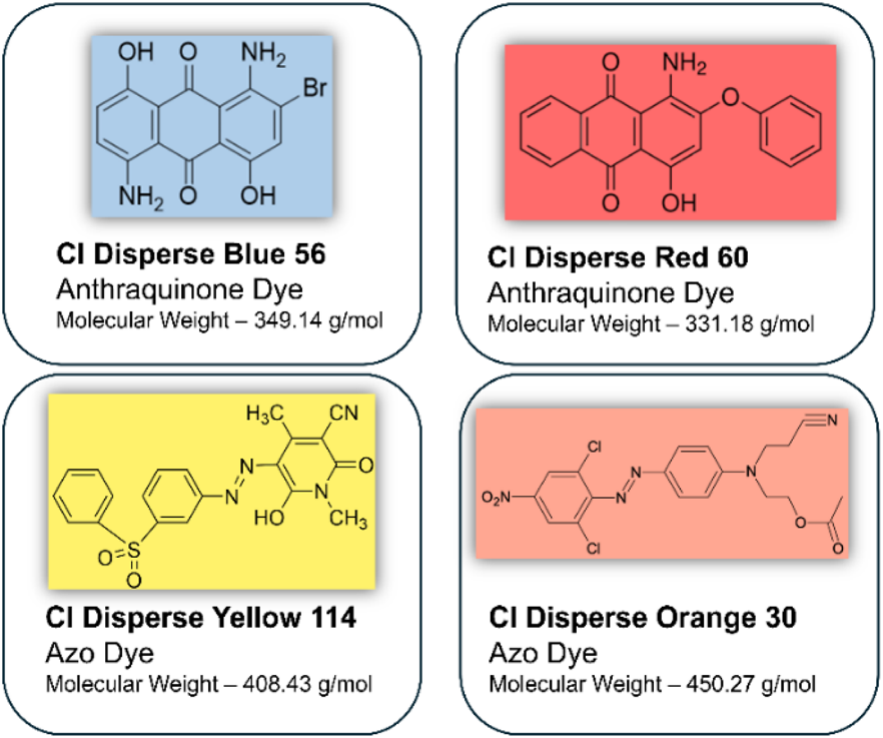
Azo and anthraquinone disperse dyes experimented.

White PET fabric substrates were systematically dyed using four structurally distinct disperse dyes to establish a comprehensive sample set for subsequent extraction studies. Two anthraquinone-based chromophores (DB and DR) and two monoazo disperse dyes (DY and DO) were selected to encompass the characteristic molecular weight distribution of disperse dyes, which typically ranges up to 600 g mol^−1^.^30^ The molecular structures and corresponding molecular weights of the selected dyes are presented in Fig. 2.

**Fig. 3:**
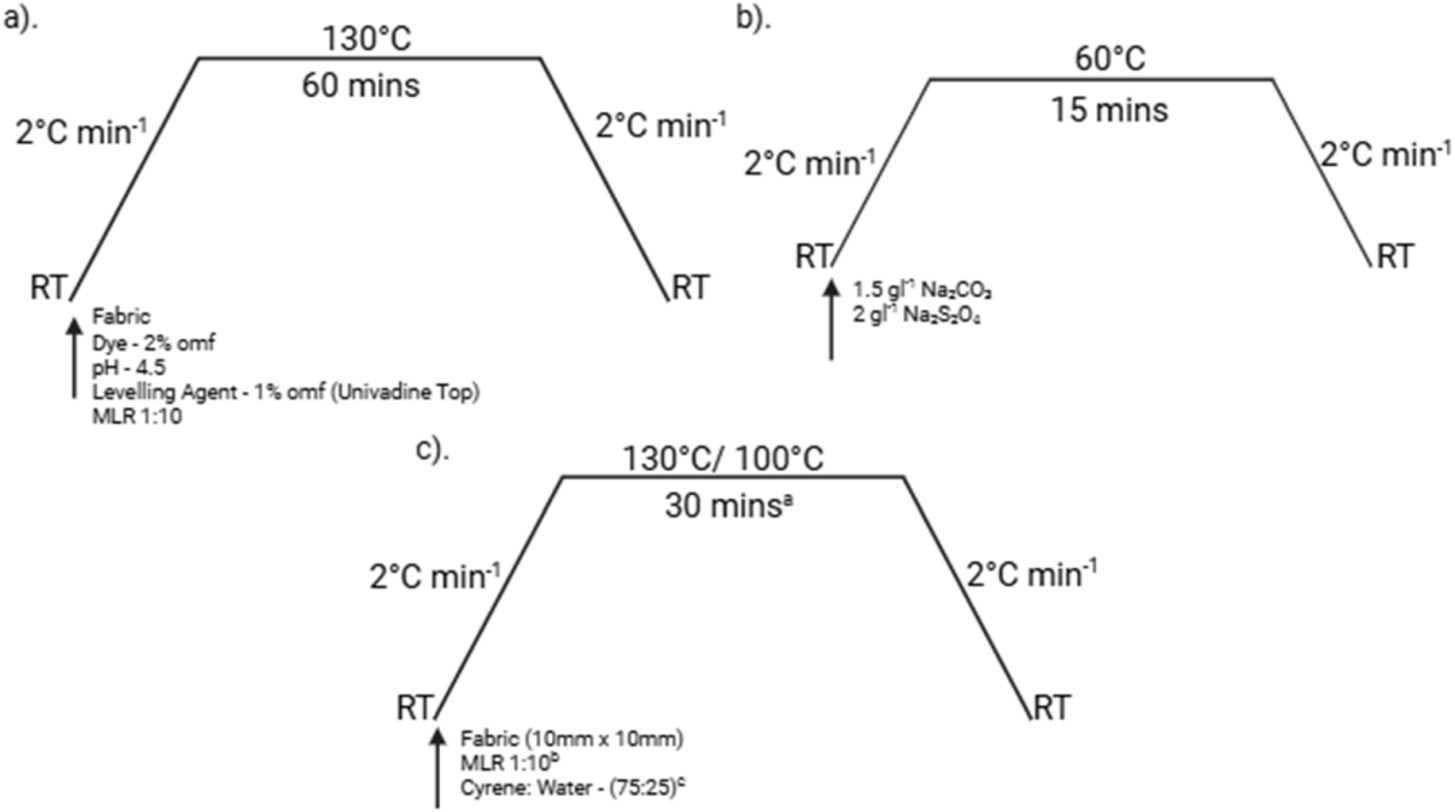
Overview of treatment profiles: (a) dyeing profiles showing applied conditions and chemical structures of the dyes; (b) reduction clearance profiles with associated treatment conditions; and (c) dye removal profiles indicating the chemicals used and the corresponding process conditions. (a) The optimum extraction time was identified. (b) The optimum material-to-liquor ratio (MLR) was determined as 1 : 10, corresponding to a fabric loading of 10%. (c) The optimum solvent composition was established as 75% Cyrene and 25% water (w : w).

Dyeing operations were performed under high temperature, high pressure (HTHP) conditions at 130 °C (Fig. 3a) using 40 g fabric specimens in 1000 cm^3^ stainless steel dye vessels with a material-to-liquor ratio (MLR) of 1 : 10. The dye bath pH was maintained at 4.5 through implementation of an acetic acid–sodium acetate buffer system to optimise dye–fibre interactions and minimise hydrolytic degradation. Following the dyeing process, fabric specimens were subjected to warm water rinsing procedures and three consecutive reduction clearing cycles at 60 °C (1 : 10 MLR), each maintained for 15 minutes (Fig. 3b). The reduction clearing protocol was continued until the rinse effluent achieved complete visual clarity, thereby confirming the quantitative removal of surface-deposited and unfixed dye particles.

Post-dyeing treatment encompassed soaping with Ultravon JUN (2 g l^−1^) surfactant at 40 °C for 10 minutes to eliminate residual auxiliaries, followed by sequential warm and cold-water washing cycles. Dyed fabric specimens were subsequently air-dried at ambient temperature to prevent thermal-induced structural modifications of the chromophore-fibre matrix.

#### 2.2.2. Repeated extraction of dye under pre-identified optimum conditions

This experimental phase was designed to evaluate the efficacy of previously identified optimal extraction parameters, specifically temperature, Cyrene-to-water (C : W) ratio, treatment duration, and fabric loading, as established through preliminary optimisation studies reported in the literature.^31^ Dyed fabric samples (3 g each) were subsequently subjected to treatment with a Cyrene/water mixture (75 : 25 w/w), in accordance with the temperature and time profile depicted in Fig. 3C. Dye extraction procedures were performed in triplicate under established optimal conditions across three consecutive treatment cycles to assess the cumulative extraction efficiency achievable through iterative processing. Additional experiments were performed at 100 °C to assess the temperature dependence of the extraction mechanism and evaluate process flexibility under reduced thermal conditions. This evaluation was performed over a single treatment cycle. Post-treatment characterisation of fabric specimens was performed through spectrophotometric analysis to quantify residual dye content on the fabric surface *via* colour strength determination. Reflectance measurements were obtained using a Datacolor Spectra 1000 series spectrophotometer, with colour strength values calculated using the Kubelka–Munk equation:1
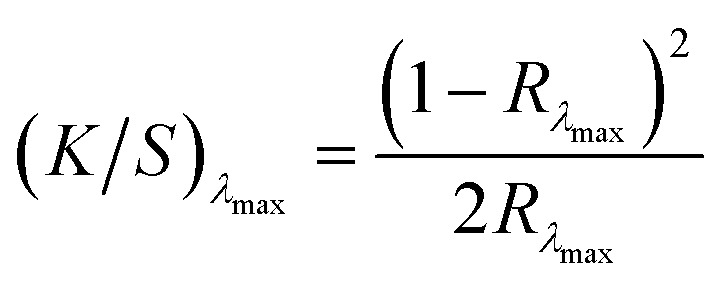
where *K* represents the absorption coefficient, *S* denotes the scattering coefficient, and *R* corresponds to the fabric reflectance at the wavelength of maximum absorption (*λ*_max_). The maximum absorption wavelengths employed for spectrophotometric analysis were DB-630 nm, DR-520 nm, DY-430 nm, and DO-450 nm.

Relative colour strength retention (*C*%) was calculated at *λ*_max_ according to:2
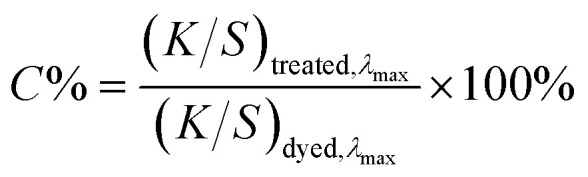


The percentage colour reduction achieved through extraction treatment was subsequently determined using:

3 Percentage of colour reduction = (*C*_*a*_ − *C*_*i*_)%

where *C*_*a*_ is the colour strength of the dyed sample and *C*_*i*_ is the colour strength after (*i*) dye removal cycles (*i* = 1, 2 or 3).

#### 2.2.3. Statistical analysis (ANOVA) and Turkey comparison

Statistical evaluation of the experimental data was performed using two-way factorial analysis of variance (ANOVA) through Minitab software version 22.3 to assess the significance of main effects and their interaction on dye extraction efficiency. The statistical model incorporated three components: dye type as Factor A (four levels: CI Disperse Blue 56, CI Disperse Red 60, CI Disperse Yellow 114, and CI Disperse Orange 30), treatment cycle as Factor B (three levels: cycles 1, 2, and 3), and their interaction term (dye × cycle). The response variable was defined as the percentage reduction in colour strength derived from *K*/*S* values.

To identify specific differences between groups, Tukey's Honest Significant Difference (HSD) post hoc test was subsequently applied.^32^ This test was carried out independently for the dye variable to assess whether dye removal efficiency differed significantly between the anthraquinone and azo dye systems. It was also used to compare the percentage reduction in colour strength across the three treatment cycles. This statistical approach allowed for a systematic assessment of the individual and combined effects of the experimental factors on the solvent-mediated removal of disperse dyes.

#### 2.2.4. Analysis of topological properties of dye molecules

The two-dimensional structures of the four selected disperse dyes were first constructed using ChemDraw (version 25). These structures were subsequently converted into three-dimensional models and subjected to energy minimisation in Chem3D employing the MM2 force field. The lowest-energy conformations obtained from this process were used for further analysis. This method is utilised due to the fundamental importance of molecular descriptors in facilitating comprehensive cheminformatic and chemometric analyses.^33,34^ From the optimised structures, a range of molecular descriptors was calculated, including Connolly's molecular area (CMA), Connolly's accessible area (CAA), Connolly's solvent-excluded volume (CSEV), molecular ovality, *etc.* These calculations were performed using a water probe with a radius of 1.4 Å to approximate solvent accessibility. Additional chemical and topological parameters were also derived from the energy-minimised structures. These included topological diameter, polar surface area, Wiener index, and the number of hydrogen bond donors and acceptors. To support visual interpretation, each dye molecule was rendered to display front, top, and side orientations.

#### 2.2.5. Correlation study on the topological and chemical properties

Following the modelling of chemical structures and the identification of topological and chemical descriptors for the molecules, a Pearson and Spearman correlation analysis was carried out to explore potential relationships between molecular characteristics and dye removal efficiency.^35,36^ This analysis aims to visualise and better understand how these descriptors influence the removal of dyes, and to examine the role played by molecular complexity, size, and shape.

## 3 Results and discussion

### 3.1 Multiple cycle dye extraction of disperse dyed PET fabrics

**Fig. 4:**
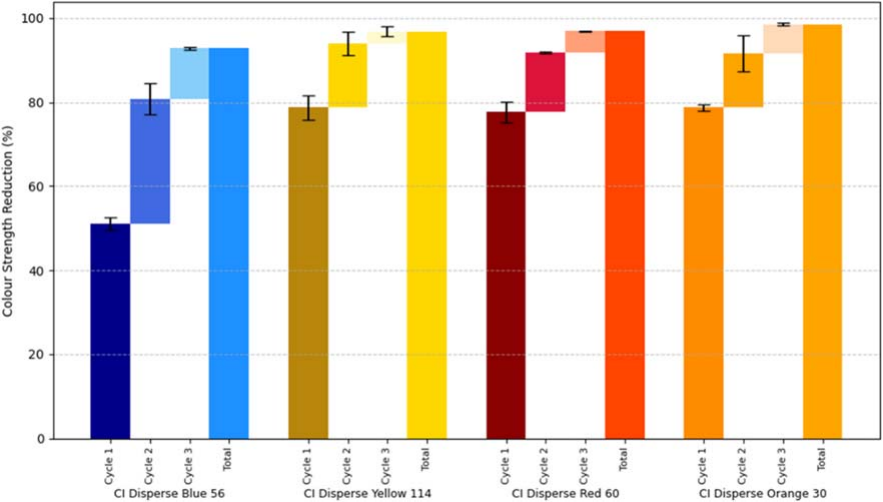
Colour strength reduction percentages up to 3 cycles under optimum conditions identified.

The percentage reduction in colour strength was evaluated across three consecutive treatment cycles carried out under optimised conditions (Fig. 4). All tested dyes exhibited removal efficiencies exceeding 92%, with specific values of 92.7% for DB, 96.9% for DR, 96.8% for DY, and 98.5% for DO. These results indicate that treatment at 130 °C significantly enhances dye extraction, likely due to thermal swelling of the PET fibres, increased molecular mobility, and accelerated reaction kinetics associated with elevated thermal energy.^37,38^ The consistently high removal rates across structurally diverse dyes support the effectiveness of the optimisation strategy and its applicability to both azo and anthraquinone dye classes.

**Fig. 5:**
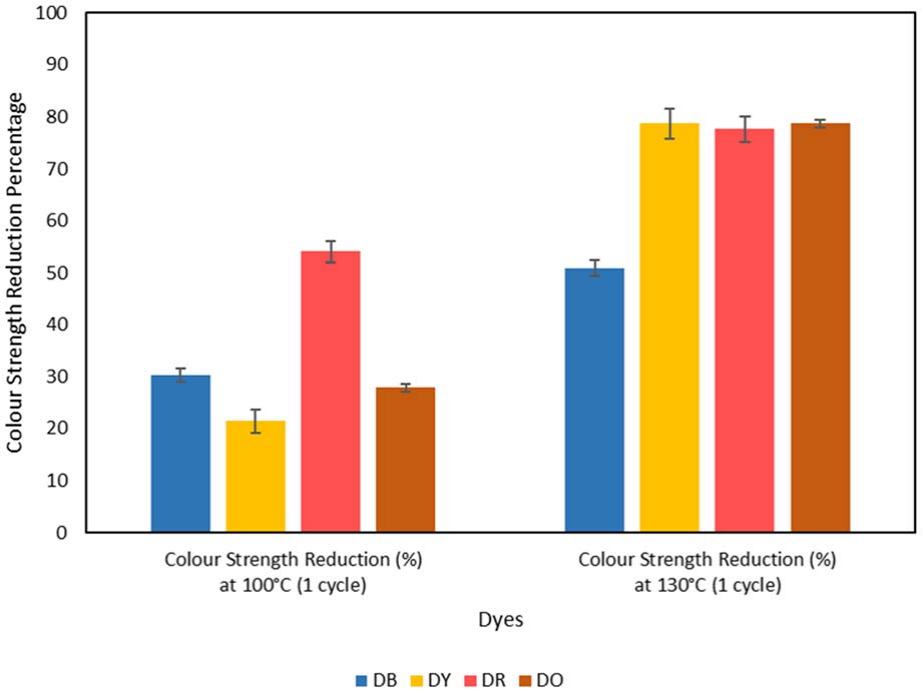
Percentage reduction in colour strength of dyed polyester fabrics following dye removal at 100 °C and 130 °C. Common treatment conditions included a C : W ratio of 75 : 25, a treatment duration of 30 minutes, and a fabric loading of 10%.

Fig. 5 compares the colour strength reduction following single-cycle treatments at 100 °C and 130 °C, using identical processing parameters (carrier-to-water ratio 75 : 25, treatment time 30 min, and fabric loading 10%). At 130 °C, DB showed a removal efficiency of 50.9%, markedly lower than DR (77.7%), DY (78.8%), and DO (78.7%). This disparity prompted further investigation into the influence of molecular properties on dye removal.

The increase in temperature from 100 °C to 130 °C resulted in substantial improvements in removal efficiency: DB increased by 68%, DR by 43%, DY by 268%, and DO by 182%. At the lower temperature, smaller dye molecules such as DB and DR exhibited relatively higher removal than the larger DY and DO, likely due to limited fibre swelling and restricted solvent penetration. In contrast, at 130 °C, enhanced fibre porosity allowed molecular characteristics such as topology and chemical structure to exert greater influence on the extraction process.^39^ These observations suggest that elevated temperature not only facilitates physical diffusion but also enables more complex dye–solvent interactions, particularly for structurally intricate dye molecules.

### 3.2 ANOVA analysis and Turkey honest difference study

**Fig. 6:**
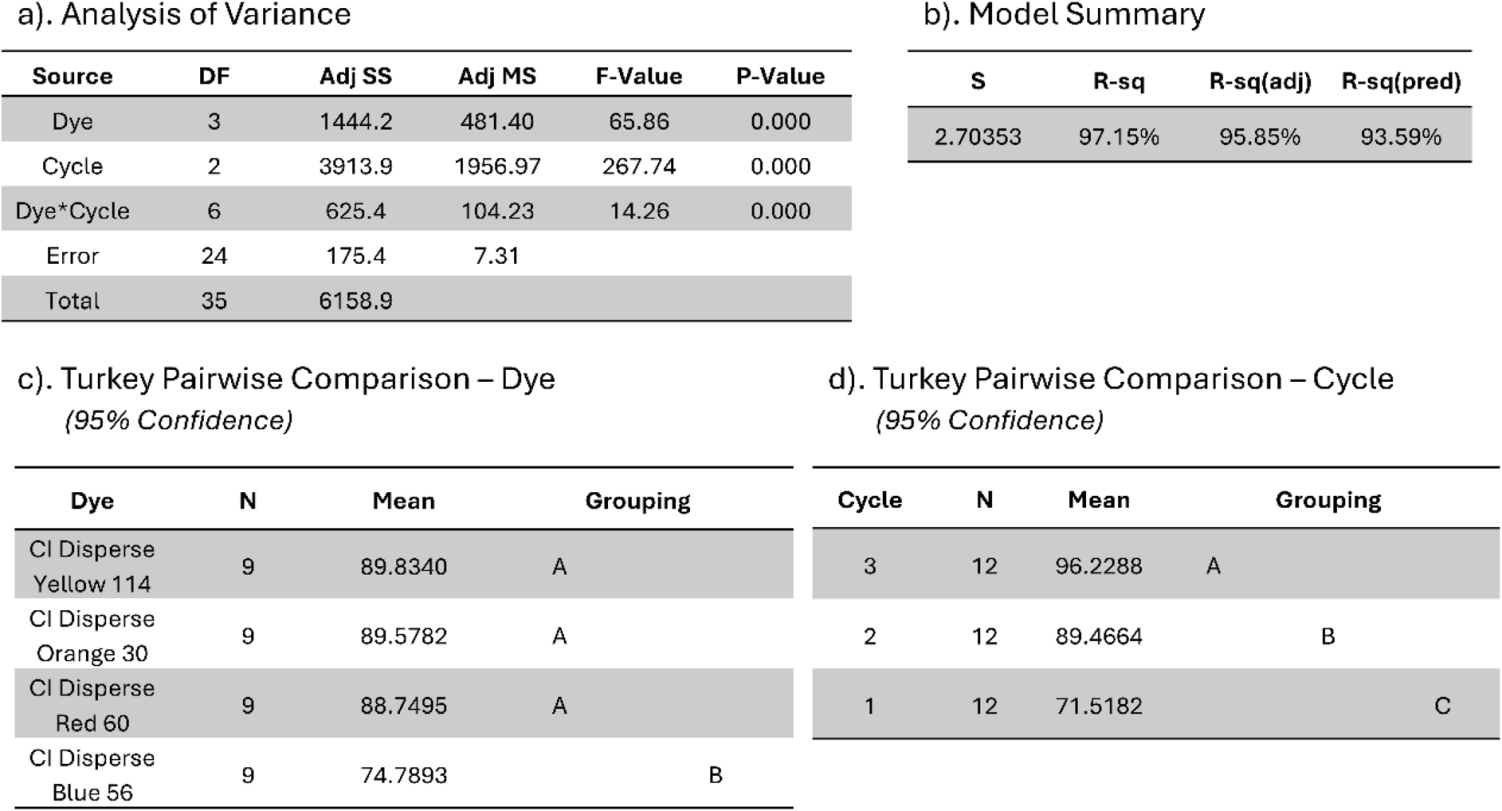
Results obtained from the ANOVA analysis and Turkey pairwise comparison.

The ANOVA revealed statistically significant effects for all examined factors on dye removal efficiency (Fig. 6a). The main effect of dye type was highly significant (*F*_3_,_24_ = 65.86, *p* < 0.001), indicating that different dyes exhibited varying degrees of removal efficiency. Similarly, the number of treatment cycles demonstrated a highly significant effect (*F*_2_,_24_ = 267.74, *p* < 0.001), suggesting that repeated treatments substantially influenced dye removal performance. The interaction between dye type and treatment cycles was also statistically significant (*F*_6_,_24_ = 14.26, *p* < 0.001), demonstrating that the effectiveness of multiple treatment cycles varied depending on the specific dye being treated. The model fit (*R*^2^ = 97.15%) provides confidence in the statistical relationships identified and supports the reliability of these findings for predicting treatment outcomes under similar experimental conditions (Fig. 6b).

Turkey's post hoc analysis revealed distinct patterns in treatment effectiveness that provide insights into the underlying removal mechanisms. Regarding dye-specific responses, three dyes (DY, DO and DR) showed statistically equivalent removal efficiencies, with mean values of 89.83%, 89.58%, and 88.75% respectively (Fig. 6c). However, DB exhibited significantly lower removal efficiency (74.79%) compared to the other three dyes. The similar removal efficiencies observed for DY, DO, and DR suggest these dyes possess comparable molecular characteristics that facilitate extraction, potentially related to the structures or molecular dimensions that enable effective solvent interaction.

For treatment cycles, all three cycles differed significantly from one another (*p* < 0.05), with mean removal efficiency increasing progressively from 71.52% after the first cycle to 89.47% after the second cycle, reaching 96.23% after the third cycle. This sequential improvement demonstrates cumulative treatment benefits and suggests that the removal process follows a kinetic pattern where initial treatments remove readily accessible dye molecules, whilst subsequent cycles target more tightly bound or structurally embedded dye particles (Fig. 6d). The interaction suggests that whilst some dyes respond progressively to increased treatment cycles, others may reach removal plateaus earlier, indicating different thermodynamic and kinetic limitations for each dye type.

### 3.3 Dye molecule topology on dye extraction efficiency

In this study, the relationship between the topological and chemical descriptors of four different dyes, modelled using ChemDraw software, was evaluated to understand their influence on dye removal performance. All other conditions, including the substrate (PET fabric), the solvent (Cyrene), and the dye removal process conditions, were constants for all four dyed fabrics. This ensured that any differences observed in dye removal efficiency could be attributed primarily to the intrinsic structural and chemical properties of the dyes. It should be noted that this approach does not suggest that combined effects such as dye–solvent interactions or dye-process dynamics are negligible. Instead, for the scope of this investigation, these factors were assumed to remain consistent across all experiments, thereby allowing a focused analysis of how variations in dye topology and chemical characteristics govern removal performance.

**Fig. 7:**
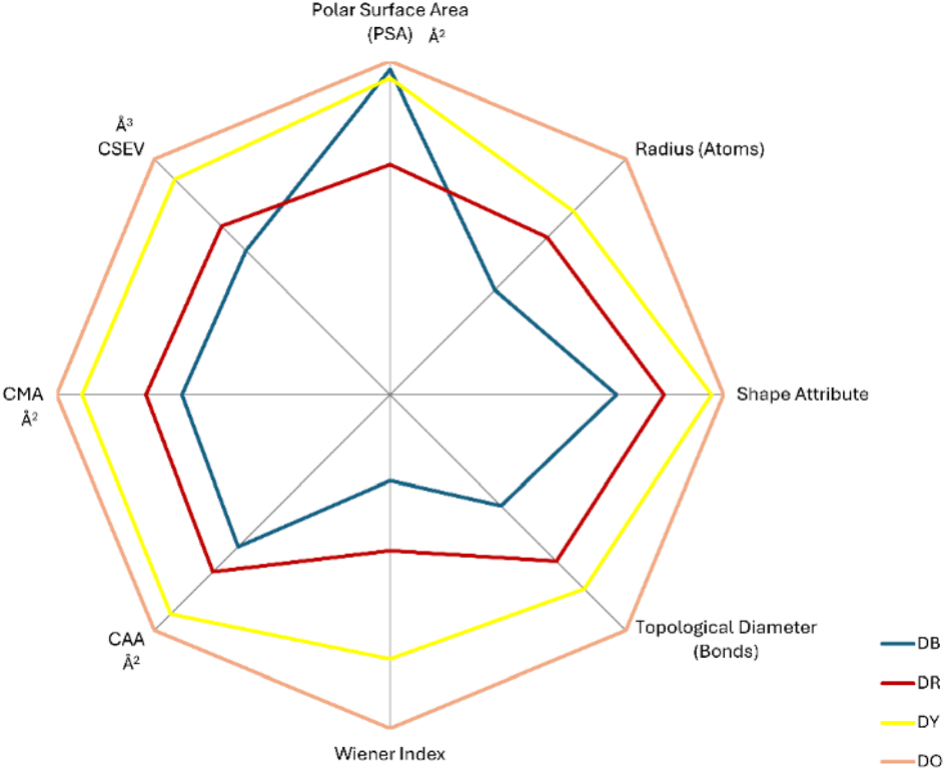
Radar plot illustrating the topological descriptor values of the four selected disperse dyes, including molecular size, molecular area, and molecular volume, to enable comparative visualisation of their structural characteristics.

The topological analysis encompassing radius, topological diameter, PSA, CMA, CAA, CSEV, Wiener index, and shape attribute (Fig. 7) consistently identifies DB as the smallest dye across all descriptors, with DR, DY, and DO exhibiting progressively larger dimensions.

This size hierarchy directly correlates with extraction efficiency, with the notable exception of polar surface area, which shows no relationship with removal performance. The absence of polar surface area correlation is particularly significant because polar surface area quantifies the molecular regions capable of electrostatic interactions and hydrogen bonding with solvents. Since dyes with varying polar surface areas exhibit similar extraction efficiencies regardless of their capacity for polar interactions, this indicates that electrostatic interactions and hydrogen bonding are not the primary driving forces for dye extraction in the Cyrene system. Instead, extraction efficiency depends on spatial geometry and non-polar interactions, where increased molecular dimensions provide greater surface area for van der Waals interactions and improved solvation shell formation. The consistent ranking across multiple topological descriptors demonstrates that molecular size and shape complementarity between the compact Cyrene solvent and the expanded dye structures governs the thermodynamic favourability of the extraction process, with larger dyes offering more extensive interaction sites for solvent association through predominantly dispersion forces rather than polar interactions.

Molecular surface properties exhibit consistent relationships with extraction efficiency, demonstrating a clear structure–activity correlation. The Connolly accessible surface area (CAA) increases progressively across the dye series: DB (430.9 Å^2^), DR (502.1 Å^2^), DY (622.1 Å^2^), and DO (668.5 Å^2^), compared to Cyrene's smaller surface area of 270.6 Å^2^, providing enhanced interaction sites for van der Waals forces. Correspondingly, the Connolly molecular area (CMA) follows an analogous trend: DB (231.8 Å^2^), DR (272.0 Å^2^), DY (343.1 Å^2^), and DO (371.5 Å^2^), representing the van der Waals surface available for direct dye–solvent contact, whilst the Connolly solvent excluded volume (CSEV) demonstrates the same proportional increase: DB (200.1 Å^3^), DR (233.9 Å^3^), DY (299.1 Å^3^), and DO (327.5 Å^3^). These three geometric descriptors collectively indicate that larger molecular architectures create more extensive dye–solvent interfaces, facilitating stronger cumulative intermolecular interactions through increased contact area and optimised molecular packing geometries. The linear correlation between all surface parameters and extraction efficiency suggests that the enhanced solvation of larger dyes results from the favourable size complementarity between the compact Cyrene molecules and the expanded surface topology of the higher molecular weight disperse dyes. This interaction allows for more effective molecular recognition and solvent–solute association through maximised interfacial contact.

Notably, the polar surface area data do not correlate with extraction efficiency: DB (126.6 Å2, 50.9% extraction), DR (89.6 Å2, 77.7% extraction), DY (123.2 Å2, 78.8% extraction), and DO (129.9 Å2, 78.7% extraction). This absence of correlation indicates that polar surface area is not a determining factor in the extraction process. Instead, the data indicate that molecular dimensions, structural complexity, and lipophilicity govern extraction efficiency. In the 75 : 25 Cyrene–water system, extraction occurs predominantly through hydrophobic interactions with the Cyrene phase, while the aqueous component functions mainly as a medium modifier rather than an active extraction agent. It is important to note that a larger molecular size does not necessarily imply greater structural complexity, and structurally complex dyes are not inherently of higher molecular weight. A larger molecular size can enhance solvent interaction by increasing the available surface area, whereas structurally complex dyes often create more voids within their architecture, enabling greater solvent penetration and facilitating dye–solvent interactions. In contrast, tightly packed, planar, low-molecular-weight dyes present fewer accessible sites, limiting solvent association.

### 3.4 Correlation between dye removal and modelled dye molecule descriptors

#### 3.4.1. Influence of dye molecular dimensions on removal efficiency

**Fig. 8:**
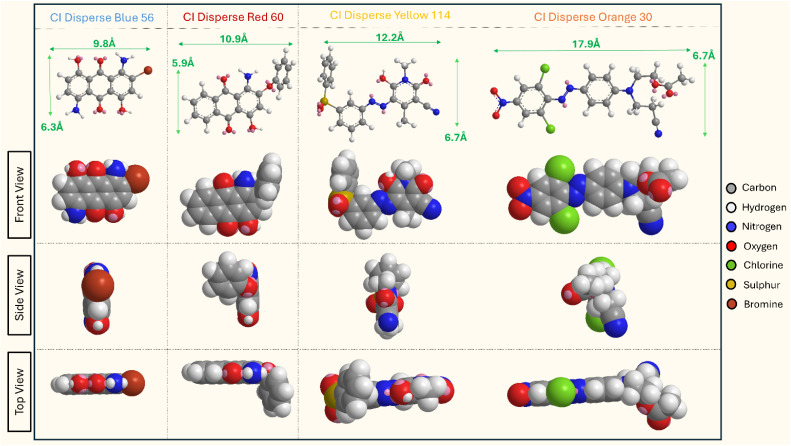
Modelled 3d structures of four disperse dyes.

Examination of the dye molecular structures (Fig. 8) reveals significant variations in molecular dimensions that correlate with extraction efficiency. The molecular lengths demonstrate a progressive increase across the series: DB dye (9.8 Å), DR dye (10.9 Å), DY dye (12.2 Å), and DO dye (17.9 Å). Analysis of molecular projections indicates that DB dye has a relatively planar, structurally simple configuration compared to the three-dimensional complexity exhibited by the other three dyes.

The enhanced extraction percentages observed for the structurally complex dyes (DR, DY, and DO) compared to the planar DB dye can be attributed to fundamental thermodynamic principles governing solute–solvent interactions. Dyes with larger molecular surface areas and volumes present increased opportunities for intermolecular interactions with the Cyrene extraction solvent through van der Waals interactions, dipole–dipole interactions, and potentially hydrogen bonding. According to principles of physical chemistry, the Gibbs free energy of solvation becomes more favourable as the number of potential interaction sites increases, thereby promoting greater extraction efficiency.

Whilst literature indicates that smaller dye molecules typically exhibit faster diffusion kinetics during dyeing, studies demonstrate that at extended dyeing times (60 minutes), both small and large dye molecules achieve complete penetration to the fibre core.^40^ Therefore, differences in penetration depth do not constitute a limiting factor in the extraction process under the experimental conditions employed.

The superior extraction performance of structurally complex dyes can be rationalised through extraction thermodynamics, where the partition coefficient between the textile phase and solvent phase is influenced by the relative affinity of the dye molecules for each phase. The enhanced solvent–dye interactions observed with the more complex molecular structures shift the equilibrium towards the solvent phase, resulting in improved extraction yields.^37,38^

#### 3.4.2. Correlation study

The computational analysis using Chem3D reveals distinct relationships between molecular descriptors and single-cycle dye extraction efficiency from PET textiles. After one extraction cycle, DB achieved 50.9% colour reduction, whilst DR, DY, and DO exhibit significantly higher extraction efficiencies of 77.7%, 78.8%, and 78.7%, respectively.

**Fig. 9:**
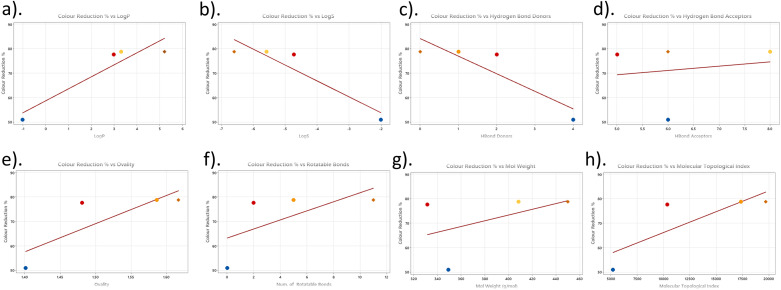
Correlation analysis between molecular descriptors of dyes and removal efficiency. Scatter plots showing the relationship between dye removal efficiency (%) and various molecular descriptors of four dyes.

The lipophilicity parameters demonstrate a clear correlation with extraction performance, with log *P* values (Fig. 9a) increasing progressively from DB (−1.02) to DO (5.21). The extraction medium comprises a 75 : 25 Cyrene–water mixture, where Cyrene (log *P* = −0.44) undergoes keto–enol tautomerisation to form the geminal-diol, creating a dynamic equilibrium between the ketone and diol forms.^25^ This aqueous system enhances the solvent's polarity and hydrogen bonding capacity, positioning it as an effective medium for solvating diverse dye structures. The enhanced hydrophobic character in the larger dyes facilitates stronger interactions with the Cyrene component, whilst the aqueous phase contributes to overall solvation through polar interactions. Correspondingly, log *S* values become increasingly negative (DB 56: −2.00 to DO 30: −6.60), indicating reduced aqueous solubility that thermodynamically favours partitioning into the organic-rich phases of the Cyrene–water system (Fig. 9b).

The hydrogen bonding capacity (Fig. 9c and d) varies across the dyes, with acceptor counts of 6, 5, 8, and 6 for DB, DR, DY, and DO, respectively, whilst donor counts decrease from 4 to 0.

Molecular flexibility significantly influences extraction performance. The ovality parameters increase from 1.40 to 1.62, suggesting that elongated, non-spherical molecular geometries correlate with enhanced extraction (Fig. 9e). The number of rotatable bonds increases from 0 in DB to 11 in DO, indicating enhanced conformational freedom that allows better geometric complementarity with solvent molecules (Fig. 9f). This flexibility potentially facilitates more favourable entropy of mixing and reduces the free energy of solvation.

Topological complexity parameters demonstrate substantial increases with extraction efficiency. The Molecular Topological index/Schultz index (Fig. 9g) ranges from 5184 for DB to 19 711 for DO, whilst the Wiener index increases from 766 to 2972. These parameters quantify molecular complexity and branching, indicating that structurally elaborate dyes exhibit superior extraction behaviour.

The relationship between molecular topology and dye removal efficiency reveals important structure–activity insights. Whilst molecular weight (Fig. 9h) shows no discernible correlation with extraction performance, indicating that mass alone is not a deciding factor in the extraction mechanism, both molecular radius and topological diameter demonstrate clear trends that influence solvent–dye interactions.

The computational analysis reveals that extraction efficiency is primarily governed by lipophilicity, molecular surface area, and structural complexity, with the 75 : 25 Cyrene–water system providing an optimal balance of hydrophobic and hydrophilic interactions. The enhanced extraction of DR, DY, and DO results from their increased hydrophobic character, enabling stronger interactions with Cyrene, expanded surface areas for intermolecular contact, and greater molecular flexibility, facilitating optimal solvent–solute geometry. The geminal-diol formation in the aqueous medium enhances the system's ability to solvate polar regions of the dye molecules whilst maintaining sufficient hydrophobic character for effective extraction. These findings align with extraction thermodynamics principles, where the mixed solvent system creates favourable enthalpy and entropy changes that drive partitioning of structurally complex dyes from the PET matrix.^4,37,38^

Dye removal experiments were conducted on 100% polyester (PET) fabrics at two temperatures, 100 °C and 130 °C. The corresponding colour strength reductions are summarised in Table 1. To investigate potential relationships between dye removal efficiency and molecular characteristics, correlation analyses were performed using four commonly applied disperse dyes representing the azo and anthraquinone structural classes, with molecular weights ranging from 300 to 500 g mol^−1^. Pearson and Spearman correlation coefficients were calculated to assess associations between dye removal percentages and the dyes' topological and chemical descriptors. No correlations were found to be statistically significant (*p* > 0.05), which is likely attributable to the limited sample size (*N* = 4) and the consequent reduction in statistical power. Both Pearson's and Spearman's analyses yielded comparable trends across the molecular descriptors, indicating consistency between linear and rank-based approaches (Table 1). Although relatively high correlation coefficients were observed for certain descriptors (*e.g.* log *P* and log *S*), none showed a strong or statistically significant association with dye removal efficiency. While these findings do not provide definitive evidence of a relationship, they offer preliminary insights and highlight the need for further research using a broader and more structurally diverse set of dyes to enable more robust statistical evaluation.

**Table 1:** Correlation coefficients (Pearson's and Spearman's) between dye removal percentage and modelled molecular descriptors (*n* = 4)

Descriptor	Pearson's correlation (*r*)	Spearman's correlation (*ρ*)	Trend
Molecular weight	0.466	0.6	Positive
Log *P*	0.934	0.8	Positive
Log *S*	−0.933	−0.8	Negative
Hydrogen bond acceptors	0.161	0.6	Positive
Hydrogen bond donors	−0.893	−0.8	Negative
Ovality	0.830	0.8	Positive
Num rotatable bonds	0.647	0.8	Positive
Molecular topological index (Schultz index)	0.822	0.8	Positive

## 4 Conclusions

The efficacy of the developed method was empirically examined through three successive treatment cycles across a range of dye types, including both azo and anthraquinone dyes with molecular weights spanning 300–500 g mol^−1^. The results demonstrated consistently high performance, with colour reduction percentages exceeding 92% for all tested dyes, and the highest achievement reached 98.5% colour reduction after three Cyrene based treatment cycles. This performance consistency across different dye chemistries indicates the robustness and versatility of the developed process. Analysis of variance (ANOVA), followed by Tukey's pairwise comparison, revealed that the individual effects of dye type, treatment cycle, and their interaction (dye × cycle) all exert a statistically significant influence on dye removal percentages. Turkey analysis shows 3 out of 4 dyes have similar means of colour reduction and DB, the least complicated planar dye molecule, has the least mean of removal efficiency. Also, each dye removal cycle has a significant effect on the colour reduction. A comprehensive correlation analysis was conducted using computationally modelled values to investigate potential relationships between dye removal efficiency and various dye properties, including structural, chemical, and physical characteristics derived from molecular modelling.

Topological descriptor analysis of the dye molecules indicates that DB possesses the smallest dimensions, surface area, and volume, followed by DR and DY. At the same time, DO is identified as the largest and most structurally complex among the four dyes. The correlation analysis of dye chemical properties did not provide significant insight regarding the dye removal, but scatter plots and correlation coefficients are presented here, which require a higher number of dye types to yield better results. However, it was observed that some descriptors, such as log *P* and log *S*, have comparatively higher correlation coefficient values. Selection of descriptors was guided by both the capabilities of the chosen tool and the necessity to include those most critical for the specific application. Although no definitive relationships were established between specific dye properties and colour reduction percentages, the analysis of structural descriptors indicated that dyes with more complex molecular shapes and larger surface areas may demonstrate enhanced interactions with the solvent system, potentially promoting more efficient removal from the fibres under the established optimal conditions.

## Author contributions

Philip Fernando: conceptualisation, data curation, formal analysis, investigation, methodology, visualisation, writing – original draft. Andrew Hebden: formal analysis, methodology, validation, writing – review & editing. Chenyu Du: funding acquisition, project administration, resources, supervision. Parikshit Goswami: funding acquisition, project administration, resources, supervision, writing – review & editing.

## Conflicts of interest

There are no conflicts to declare.

## Supplementary Material

RA-016-D5RA08950F-s001

## Data Availability

The data that support the findings of this study are available from the corresponding author upon reasonable request. Supplementary information (SI) is available. See DOI: https://doi.org/10.1039/d5ra08950f.
