# Supplementary material for: Solvent-mediated extraction of disperse dyes from polyester: correlating Cyrene extraction yields with molecular topological and chemical descriptors
Source: RSC Adv. 2026 Jan 15;16(4):3338–48. doi: 10.1039/d5ra08950f (PMC12805328; doi:10.1039/d5ra08950f)
Supplement: RA-016-D5RA08950F-s001 [file RA-016-D5RA08950F-s001.pdf]

Supplementary Information (SI) for RSC Advances.
This journal is © The Royal Society of Chemistry 2026

Supporting Information

Solvent-Mediated Extraction of Disperse Dyes from Polyester: Correlating Cyrene Extraction Yields with Molecular Topological and Chemical Descriptors.

Philip Fernando,*[a] Andrew Hebden [a], Chenyu Du [b] and Parikshit Goswami [a]

a. Technical Textiles Research Centre, University of Huddersfield, Queensgate, Huddersfield HD1 3DH, UK.
b. School of Applied Sciences, University of Huddersfield, Queensgate, Huddersfield HD1 3DH, UK.
** Corresponding Author*

Email- Philip.fernando@hud.ac.uk

Supporting Table 1: *Colour strength values (K/S) and corresponding percentage colour reduction for CI Disperse Blue 56, CI Disperse Red 60, CI Disperse Yellow 114, and CI Disperse Orange 30 across three successive extraction cycles under optimised conditions.*

**Dye/ (λ_{max})**	**After Dyeing**		**After 1st Cycle**		**After 2nd Cycle**		**After 3rd cycle**	
	**K/S**	**Colour Strength**	**K/S**	**Colour Strength**	**K/S**	**Colour Strength**	**K/S**	**Colour Strength**
CI Disperse Blue 56 (630nm)	12.6	100	5.95	47.1	1.92	15.2	0.89	7.0
CI Disperse Red 60 (520nm)	10.6	100	2.36	22.3	0.86	8.1	0.35	3.4
CI Disperse Yellow 114 (430nm)	12.2	100	2.46	20.2	0.54	4.4	0.37	3.0
CI Disperse Orange 30 (450nm)	10.2	100	2.29	22.3	0.56	5.4	0.13	1.2

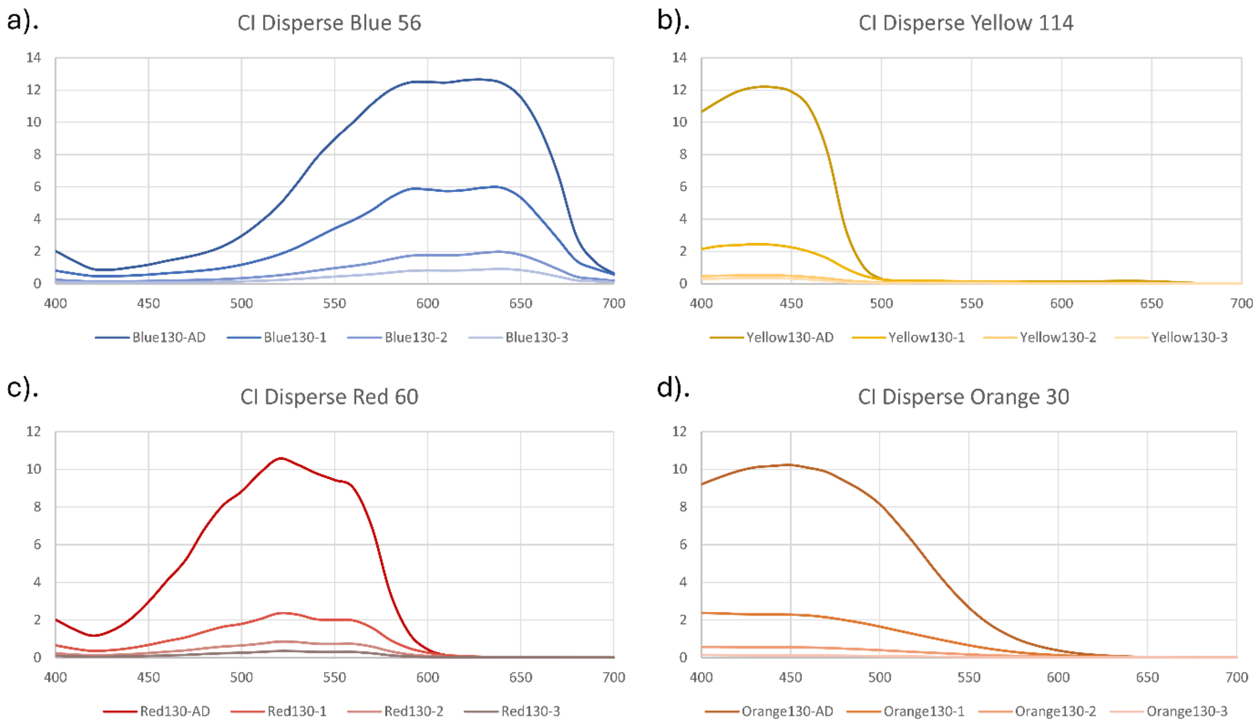

Supporting Figure 1: *Spectrophotometric colour strength (K/S) values across the visible wavelength range (400–700 nm) for CI Disperse Blue 56 (a), CI Disperse Yellow 114 (b), CI Disperse Red 60 (c) and CI Disperse Orange 30 (d) measured on dyed PET fabrics before and after solvent media.*

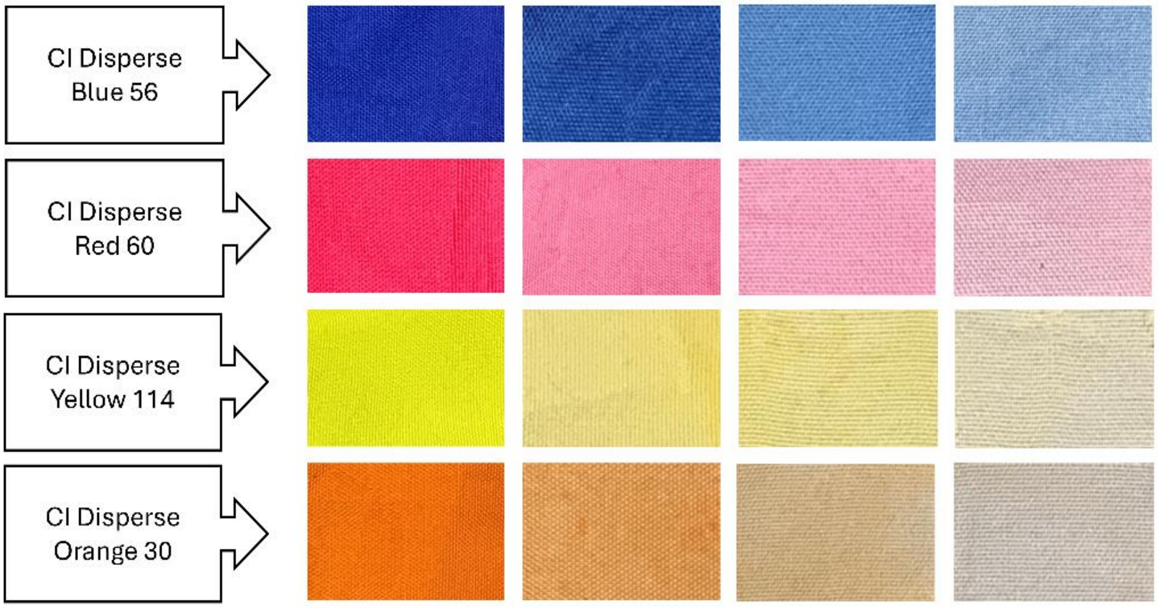

Supporting Figure 2: *Representative images illustrating progressive colour reduction of dyed PET fabrics across three successive extraction cycles using Cyrene™. From left to right: fabric appearance after initial dyeing, after the first dye removal cycle, second cycle, and third cycle under optimised treatment conditions.*

Supporting Table 2: *Computed molecular descriptors for CI Disperse Blue 56, CI Disperse Red 60, CI Disperse Yellow 114, and CI Disperse Orange 30, including Connolly molecular area (CMA), Connolly accessible area (CAA), Connolly solvent-excluded volume (CSEV), ovality, topological diameter, polar surface area (PSA), Wiener index, lipophilicity (Log P), solubility (Log S), and number of rotatable bonds, derived from Chem3D modelling of energy-minimised structures.*

Dye	***CI Disperse Blue 56***	***CI Disperse Red 60***	***CI Disperse Yellow 114***	***CI Disperse Orange 30***
Colour Reduction %	50.94	77.65	78.75	78.73
Mol Weight	349.14	331.327	408.432	450.276
LogP	-1.02371	2.97458	3.29792	5.21456
LogS	-2.0025	-4.72592	-5.58236	-6.5976
Polar Surface Area	126.64	89.62	123.19	129.86
HBond Acceptors	6	5	8	6
HBond Donors	4	2	1	0
Ovality	1.401	1.481	1.586	1.617
Num Rotatable Bonds	0	2	5	11
Connolly Accessible Area	430.906	502.061	622.068	668.542
Connolly Molecular Area	231.828	271.985	343.064	371.523
Connolly Solvent Excluded Volume	200.117	233.947	299.121	327.48
Partition Coefficient	3.57228	5.46375	2.75038	5.57559
Molecular Topological Index	5184	10354	17339	19711
Radius	4	6	7	9
Shape Attribute	19.05	23.04	27.03	28.03
Sum Of Degrees	46	56	62	62
Sum Of Valence Degrees	82	96	108.67	109.56
Topological Diameter	8	12	14	17
Wiener Index	766	1390	2356	2972

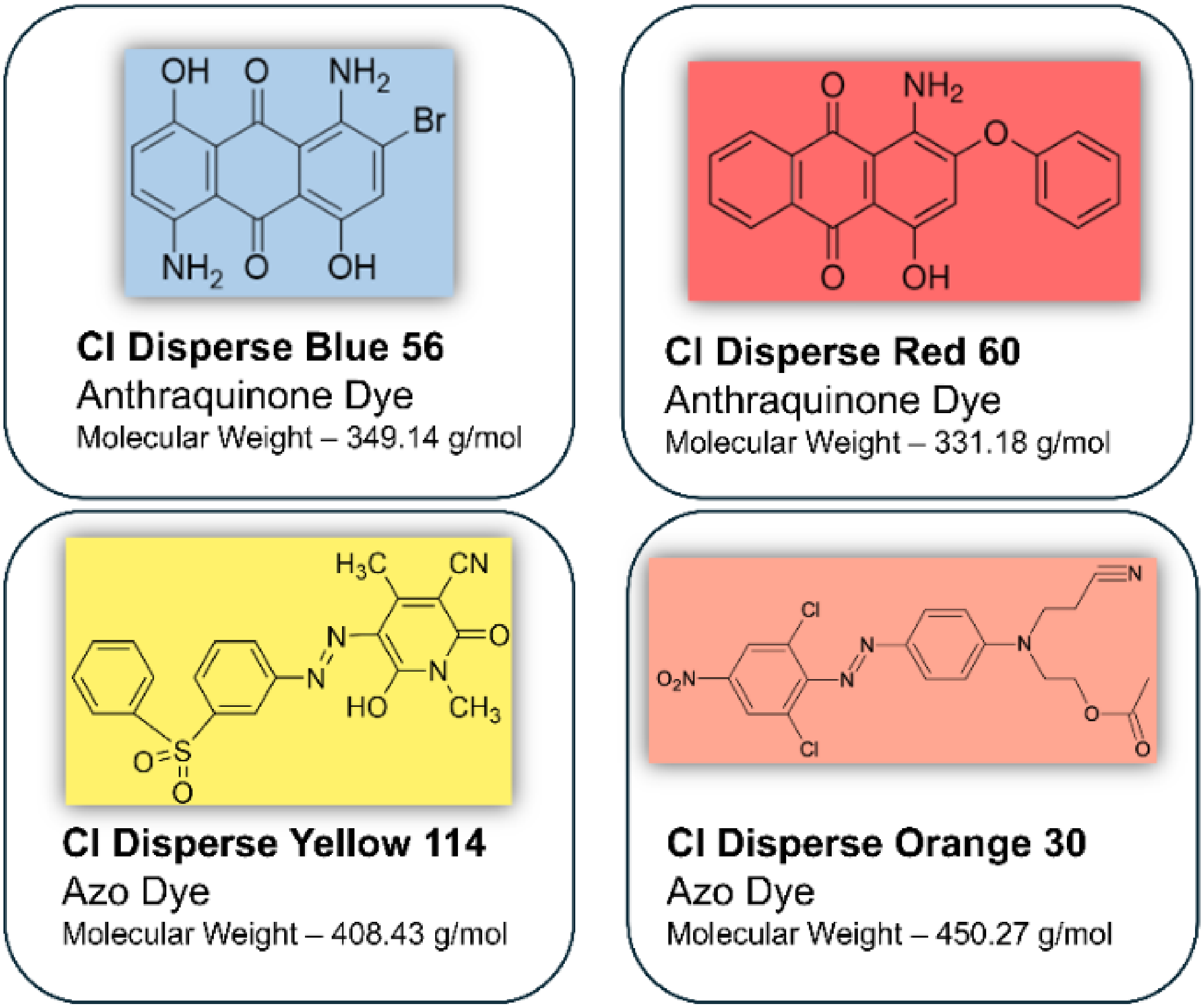

Supporting Figure 3: *Azo and Anthraquinone disperse dyes experimented.*
